# Knowledge, attitude and perceived stigma towards tuberculosis among pastoralists; Do they differ from sedentary communities? A comparative cross-sectional study

**DOI:** 10.1371/journal.pone.0181032

**Published:** 2017-07-17

**Authors:** Bezawit Temesgen Sima, Tefera Belachew, Fekadu Abebe

**Affiliations:** 1 Public Health and Medical College, Jimma University, Jimma, Ethiopia; 2 Department of Community Medicine and Global Health, Institute for Health and Society, Faculty of Medicine, University of Oslo, Blindern, Oslo, Norway; McGill University, CANADA

## Abstract

**Background:**

Ethiopia is ninth among the world high tuberculosis (TB) burden countries, pastoralists being the most affected population. However, there is no published report whether the behavior related to TB are different between pastoralist and the sedentary communities. Therefore, the main aim of this study is to assess the pastoralist community knowledge, attitude and perceived stigma towards tuberculosis and their health care seeking behavior in comparison to the neighboring sedentary communities and this may help to plan TB control interventions specifically for the pastoralist communities.

**Method:**

A community-based cross-sectional survey was carried out from September 2014 to January 2015, among 337 individuals from pastoralist and 247 from the sedentary community of Kereyu district. Data were collected using structured questionnaires. Three focus group discussions were used to collect qualitative data, one with men and the other with women in the pastoralist and one with men in the sedentary groups. Data were analyzed using Statistical Software for Social Science, SPSS V 22 and STATA.

**Results:**

A Lower proportion of pastoralists mentioned bacilli (bacteria) as the cause of PTB compared to the sedentary group (63.9% vs. 81.0%, p<0.01), respectively. However, witchcraft was reported as the causes of TB by a higher proportion of pastoralists than the sedentary group (53.6% vs.23.5%, p<0.01), respectively. Similarly, a lower proportion of pastoralists indicated PTB is preventable compared to the sedentary group (95.8% vs. 99.6%, p<0.01), respectively. Moreover, majority of the pastoralists mentioned that most people would reject a TB patient in their community compared to the sedentary group (39.9% vs. 8.9%, p<0.001), respectively, and the pastoralists expressed that they would be ashamed/embarrassed if they had TB 68% vs.36.4%, p<0.001), respectively.

**Conclusion:**

The finding indicates that there is a lower awareness about TB, a negative attitude towards TB patients and a higher perceived stigma among pastoralists compared to their neighbor sedentary population. Strategic health communications pertinent to the pastoralists way of life should be planned and implemented to improve the awareness gap about tuberculosis.

## Introduction

Tuberculosis (TB) is a major global health problem, responsible for ill health among millions of people each year [1]. In 2015 TB was one of the top 10 causes of death worldwide and the leading cause of death from an infectious disease ranking above HIV/AIDS [2]. Based on the world health organization Global Report 2016 [2], there were 10.4 million new TB cases in 2015 and 1.4 million TB deaths. Moreover, there were 0.4 million deaths from TB disease among HIV-positive people and approximately 250 000 deaths from (multi-drug resistant/resistant to rifampicin) MDR/RR -TB [2]. Most of the estimated number of cases in 2015 occurred in Asia (61%) and WHO Africa region (26%) and 80% of the HIV-positive TB cases are in Africa [2].

The incidence of TB doubled more in Africa during the last two decades [3]. Ethiopia ranked among the top 20 high TB burden countries globally and the incidence of TB in 2015 was 191 cases per 100,000 people [2].

In Ethiopia, Pastoralists are the most affected by TB [4, 5] and they depend on livestock for their survival, migrating seasonally from place to place with their animals in search of fresh pasture and water [5, 6]. Pastoralism accounts for the livelihood of 50–100 million people in developing countries and Ethiopia has the largest pastoralist population in East Africa (7–8 million) [6].

It is increasingly recognized that the limited awareness about TB and health seeking behavior among communities that favor the use of traditional healers over biomedical approaches and perceived stigma about TB make TB control activity difficult under the DOTS strategy [7–9].

There are studies conducted on pastoralist communities health care seeking behavior, Knowledge, Attitude and practice towards TB [10–12] and reported a low level of community awareness about TB, preference of visiting traditional healers and the association between visiting traditional healers and treatment delay [4, 8, 9, 13]. But to our knowledge, no comparative study has been done in the pastoralist community with their neighboring sedentary community.

Therefore, understanding the overall knowledge, attitude and perceived stigma towards TB in comparison to the sedentary community might be used to identify a behavioral gap that is specific for each population and may be used as a baseline to develop TB control strategy that is pertinent to the pastoralist way of life.

## Objectives

The main objective of this study was to assess and compare the pastoralist’s knowledge, attitude and perceived stigma towards TB and health seeking behavior to the neighboring sedentary community in the Fentale district, Eastern Ethiopia.

## Methods and materials

### Ethical consideration

This study was approved by the Norwegian Social Science Data Service (NSD) and Ethical Review Committee of Jimma University, Jimma, Ethiopia and Oromia Regional Health Office Ethical Review Committee, Addis Ababa, Ethiopia. Written informed consent was obtained from the respondents before the administration of the questionnaires and confidentiality was maintained by using a code instead of a personal identifier, and FGD participants were given nickname that they choose to be called and no personal identifier was taken.

### Study area and population

This study was conducted in Fentale (Kereyu) Wereda (equivalent to district) which is located in the East Shoa zone of Oromia, Southern part of the Northern rift valley of Ethiopia. The area falls within an altitude range of 800–1100 masl. The study area is found at a distance of 200km from the capital city of Ethiopia, Addis Ababa. The capital of the district is called Metahara. The total land area of the district is 1170 km2 with a total population of 76,367. The majority of the pastoralist populations are Nomadic but some practice Ago-Pastoralism. Pastoralists in this area migrate to a neighboring sub-districts during the dry season. The economy depends mainly on livestock production.

The district has 18 rural *kebeles* (the smallest unit of the district). The study was conducted in two comparative communities, a pastoralist community and a neighboring sedentary community in the district. The sub-districts included in this study from pastoralist were *Kobo*, *Benti*, *Dhabiti and D/Hadu while Alge* and *G/Dima* are from the sedentary community.

### Study design and sampling

A community-based comparative cross-sectional study supplemented by qualitative method focus group discussion (FGD) was conducted from September 2014—January 2015.

Before the actual data collection, the sub-districts of the pastoralist and Sedentary villages were identified with the help of Health Extension Workers and TB focal person. Simple random sampling was used to select the sub-districts. There was a total of 18 sub-districts in *the Fentale* District and three of the sub-districts were sedentary villages (two rural and one urban), using the lists of the sub-district as a sampling frame. A lottery method was used to select four of the 15 pastoralist villages and included two rural sedentary villages. The predetermined sample was distributed to each selected village proportional to the size. Then systematic sampling was used to select the households in each village and the first household was selected randomly by rolling a bottle in the center of the village and following the direction of the neck. In each household, the head of the household was selected as a respondent and in the case of having two heads in the household, existing at the same time; the lottery method was used to select one of the heads. In the case of refusal and absence in a household, the next nearest household was selected and interviewed. The same procedure was repeated to select 247 respondents from the sedentary community. Residents of the selected study sites age 18 years and above were included in the interview.

### Sample size calculation

The sample size was calculated using a single population proportion formula with the assumption of an overall TB knowledge 45.6% proportion taken from a study done in Shinile Town, Somali regional state [11] and 95% confidence interval (CI), 5% precision and 10% nonresponse rate using the following formula,

$$n=\frac{{\left(\frac{z\alpha }{2}\right)}^{2}\;x\;P(1-p)}{d^{2}}$$

Where,

n = minimum sample size
Zα/2 = 1.96 (95% CI)
P = 45.6%
d = 5% margin of error

Therefore, the value of n was calculated to be 381.

Considering 10% non-response rate, the final sample size was adjusted to 419 household heads.

A Similar sample size was taken from both groups and a total of 338 individuals among the pastoralists and sedentary villages were interviewed and 33 individuals interviewed under the sedentary community were excluded based on pre-sated exclusion criteria from the data because the socio-demographic data of those individuals indicated they were more of the agro-pastoralists.

#### Qualitative data sampling

A total of 22 individuals who did not participate in the house to house interview were involved in 3 FGDs which comprises 6–10 individuals in each discussion. One FGD was conducted with the sedentary community members, whereas two were with the pastoralist community. The participants were adults aged 18 years and above and residents of the study sites. They were selected conveniently and the homogeneity of the group was maintained in terms of sex, educational status and position in the community. The final sample size was determined based on the saturation of information.

### Data collection and measurements

Seven trained field interviewers who completed grade 12 and above and fluent in Afan Oromo (local language) collected the data using a semi-structured questionnaires adopted from the World Health Organization (WHO) sample Advocacy, communication and social mobilization, Knowledge, Attitude and Practice (KAP) survey questionnaire[14] and other similar studies-[10, 15, 16].

The tools had sections on their socio, demographic characteristics, TB knowledge, and awareness, attitude towards TB, Perceived stigma, TB information, and health seeking behavior. Three local coordinators who had experience of field work supervision were trained and involved in the supervision of the data collection process. The tools were pre-tested for reliability, understandability, and clarity of the language used in a community in the Jimma zone with similar socio-demographic characteristics.

#### Knowledge of the community towards TB

The Overall knowledge of the community toward TB was assessed using 16 questions. (1) Ever heard of TB; (2) able to mention bacteria or germ as a cause of TB; (3) able to mention the correct signs and symptoms of TB (3.cough for three weeks or more, 4.coughing up blood, 5.weight loss, 6.chest pain, 7.fever or sweating, 8.weakness); (9) know that TB can be transmitted; (10) able to list the correct mode of transmission of TB (droplets from coughing and sneezing of a person having active TB, sharing cups with a person having TB, sharing utensils); (11) knowing that anybody can be infected with TB; (12) knowing that TB is curable; (13) knowing that TB can be treated by modern drugs given by health facilities; (14) knowing that TB is preventable; (15) able to mention correct preventive methods (using separate room, covering mouth and nose when coughing and sneezing, avoiding sharing cups and not avoiding handshake) and (16) Knowing that the drugs are available for free. The correct (yes) response to each question was scored as one showing positive response and incorrect (no/I don’t know) response was scored as zero showing a negative response. Then the response to these questions were added together to generate a knowledge score from 0 to 16 (including 6 signs and symptom questions) and the overall score was dichotomized using 50% as a cutoff value and those who have 50% and above correct response to the questions were coded “1” for higher overall TB knowledge and below 50% coded “0” for low overall TB knowledge. Likewise, the scores were generated for the four subscales of knowledge about TB (causes of TB, signs and symptoms, treatment and prevention methods) and the knowledge about the signs and symptoms were categorized as low and high levels of knowledge.

#### Attitude towards Tuberculosis

Attitude of the community towards TB was assessed using 7 questions: (1) able to mention TB as a serious / somehow serious disease; (2) able to mention TB as a serious disease and problem in their community; (3) able to mention positive feelings towards the disease; (4) able to discuss TB status (Whom to talk to if find out one has TB; spouse, parent, child/children, family member, close friends, none); (5) feeling compassion and desire to help a person with TB (7) Does having TB carry the same stigma as AIDS. An answer consistent with the correct attitude towards the disease and to a person with TB was scored with one. An answer not consistent with the correct attitude towards the disease and to a person with TB was scored zero. The proportions to each response in each comparative group were used with significance level to see the difference.

#### Perceived stigma towards TB

The Perceived stigma towards TB was assessed using 11 questions. (1) if you had TB, others would think less of you; (2) if you find out you had TB, you would feel ashamed and embarrassed (3); if you find out you have TB, you would think less of you; (4) If you had TB, others would avoid you; (5) If you had TB, you would have a problem of finding a partner even after cure; (6) If you had TB, your partner would refuse to have sex with you; (7) If you had TB, you would be asked to stay away from a social group; (8) If you had TB, you would not disclose your status to anyone; (9) If you had TB, you would affect others by the disease; (10) if you had TB, others would think less of your family; (11) if you had TB, it would be a problem for your children. Each question consists of “yes” and “no” response, where ‘yes’ indicating the presence of perceived stigma and ‘no’ indicating the absence of perceived stigma. Then the responses consistent with the correct perceived stigma were scored one and the rest scored zero and some of the responses to these questions were used to generate the stigma score from 0 to 11 and the overall score dichotomized 50% as a cutoff point. Those who scored 50% and above were coded as one showing a high perceived stigma towards TB and those who scored less than 50% were coded as zero showing a low perceived stigma towards TB.

### Sources of TB information

The community sources of TB information were assessed by asking 3 questions.

(1) Where first heard about TB (HEW’s or health providers, friends and families); (2) availability of health information on TB; (3) most trusted sources of TB information (HW’s, friends/family, TV, radio, religious leaders). The correct (yes) response to each question was scored as one showing positive response and the incorrect (no) response was scored as zero showing a negative response. Then the proportions of individuals with positive and negative response were reported.

#### Healthcare seeking behavior of the community

The Healthcare seeking behavior of the respondents was assessed by four questions; (1) Communities’ frequently visited institution for general illness; (2) frequency of health care facility visit; (3) frequency of consultation to TH’ for general illness and (4) reasons for not visiting health care facilities. The proportions of individuals in each response were reported and compared among the groups.

### Qualitative data collection tool

The Focus group discussion (FGD) guide was developed based on the main thematic areas of the quantitative data tools and used to collect the qualitative data and probing was used to clarify the raised issues. The discussion was moderated by an expert who had experience on qualitative data collection and fluent in the local language and a Rapporteur participated in taking notes. An audio recorder was used to make sure no information was missed.

### Data analysis

#### Quantitative data

The data were analyzed using statistical software STATA version 13 and statistical package for social science (SPSS) version 22. The Multivariable logistic regression was used to identify the predictive variables of the overall TB knowledge and perceived stigma. The significant differences in the two group values were evaluated using a significance level of 0.05 and 95% confidence interval.

#### Qualitative data analysis

The results from the FGDs were transcribed and then translated into the English language by the same person who collected the data. Thematic areas were identified and coded and the same responses given by respondents were described and discussed.

## Results

### Socio-demographic characteristics of the respondents

A total of 338 individuals from *Kereyu* pastoralists and 247 from the neighboring sedentary community, age 18 years and above were interviewed. Thirty-one percent of the pastoralists were ranging in age from 25 to 34 years, while 33% of the sedentary group were less than 25 years of age. Most (89.9% pastoralists vs. 43.7% sedentary) cannot read and write, while 7.4% of the pastoralists and 31.6% of the sedentary group had primary education. Among the respondents, 50% of the sedentary group were farmers by occupation. However, among the pastoralists, 53.1% of the respondents were nomadic, while the remaining 38.9% were agro-pastoralists. Fifty percent of the pastoralists migrate during the dry season twice a year and almost 99% migrate within the districts. The mean migration frequency is 3.1 (2.9, 3.2) **(Table 1).**

**Table 1:** Socio-demographic characteristics of respondents from the pastoralist community and the sedentary community of Fentale district, Ethiopia.

Variables	Pastoralists, N = 338 Frequency (%)	Sedentary, N = 247 Frequency (%)	P value
Age groups			
< 25	77 (22.8)	80 (32.4)	0.02
25–34	105 (31.1.9)	77 (31.2)	
35–44	84 (24.9)	62 25.1)	
45–54	36(10.7)	12(4.9)	
55–64	20 (5.9)	10 (4.0)	
>64	16 (4.7)	6 (2.4)	
Mean age (95% CI)	35.9 (34.5, 37.4)	31.6 (30.1, 33.1)	< 0.001
Gender			
Female	265 (78.4)	133 (53.8)	< 0.001
Male	73 (21.6)	114 (46.2)	
Education			
Primary	25 (7.4)	78 (31.6)	< 0.001
Secondary	5 (1.5)	54 (21.9)	
Tertiary	4 (1.2)	7 (2.8)	
Cannot read or write	304 (89.9)	108 (43.7)	
Religion			
Christianity	0 (0.0)	125 (51.0)	< 0.001
Islam	338 (100.0)	120 (49.0)	
Ethnicity			
Oromo	338 (100.0)	88 (35.6)	< 0.001
Amhara	0 (0.0)	159 (64.4)	
Occupation			
Student	7 (2.1)	4 (1.6)	< 0.001
Business	17 (5.0)	29 (11.8)	
Civil servant	0 (0.0)	20 (8.1)	
Farmers	0 (0.0)	123 (50.0)	
Nomadic	179 (53.1)	0 (0.0)	
Agro-pastoralist	128 (38.9)	0 (0.0)	
Others	6 (1.8)	70 (28.5)	
Frequency of migration			
Once	13 (3.8)	0 (0.0)	-
Twice	169 (50.0)	0 (0.0)	
Three	34 (10.1)	0 (0.0)	
Four	81 (24.0)	0 (0.0)	
Five and more	41 (12.1)	0 (0.0)	
No movement	0 (0.0)	247 (100.0)	
Mean migration frequency	3.1 (2.9, 3.2)	0 (0.0)	
Migration destination			
Within the district	336 (99.4)	0 (0.0)	-
Out of the district	2 (0.6)	0 (0.0)	
Family members who migrate mostly			
Head of household	183	0 (0.0)	
Children	5	0 (0.0)	
All family members	150	0 (0.0)	

### Knowledge about TB

Thirty-five percent of the pastoralists and 88.7% of the sedentary group (p<0.001) first heard about TB from health care providers (HCP)/health extension workers (HEW), and 34.9% of the pastoralists and 10.9% of the sedentary group (p<0.001) first heard about TB from family members **(Table 2).** Bacilli (63.9% Vs 81%, p<0.01), Witchcraft (53.6% vgt8vs. 23.5%, p<0.01), poverty (10.9% vs. 40.1%, p<0.001), living together with untreated TB patient (67.8% vs. 93.1%, p<0.001) were mentioned as the causes of TB by the pastoralists and sedentary group, respectively **(Table 3)**. Regarding TB transmission, most (87.2% pastoralists vs. 98% sedentary group, p<0.01) mentioned that they knew how a person could get TB and (44.2% pastoralists vs. 25.9% sedentary group, p = 0.01) mentioned coughing and sneezing of a person having TB as the main route of transmission **(Table 3)**. Specific drugs provided by health care facility were mentioned as the main treatment of TB by (36.3% pastoralists vs. 75.3%, sedentary group p<0.001). Most respondants(72.5% pastoralists vs. 95.1% of the sedentary group, p<0.01) mentioned that covering the mouth and nose while sneezing and coughing as the main preventive measures against TB **(Table 3)**.

**Table 2:** Assessment of sources of information on TB among the pastoralists and sedentary communities of Fentale district, Ethiopia.

TB information source	Pastoralists	Settlers	P-value
	Frequency (%)	Frequency (%)	
Where did you first heard about TB			
Health professionals/HEWs	118 (34.9)	219 (88.7)	<0.01
Family	118 (34.9)	27 (10.9)	<0.01
Friends	85 (25.1)	30 (12.1)	0.07
Media	38 (11.2)	39 (15.8)	0.28
Others	8 (2.4)	0(0.0)	_
Trusted sources of Health information			
HEWs	224 (66.3)	215 (87.0)	<0.01
Friends/Family	27 (8.0)	9 (3.6)	0.32
Television	22 (6.5)	69 (27.9)	0.02
Radio	143 (42.3)	90 (36.4)	0.18
Religious leaders	12 (3.6)	2 (0.8)	0.41

**Table 3:** Knowledge about TB among the pastoralists and sedentary communities of Fentale district, Ethiopia.

TB knowledge assessment	Pastoralists n = 338	Settlers n = 247	P-value
	Frequency (%)	Frequency (%)	
Causes of TB			
Bacilli	216 (63.9)	200 (81.0)	< 0.01
Witchcraft	181(53.6)	58 (23.5)	<0.01
Poverty	37 (10.9)	99 (40.1)	< 0.01
Hard work	157 (46.4)	109 (44.1)	0.35
Sexual over-indulgence	116 (34.3)	85 (34.4)	0.49
Living together with untreated TB patient	229 (67.8)	230 (93.1)	< 0.01
Signs and Symptoms of TB			
Cough	274 (81.3)	194 (79.2)	0.29
Cough that lasts more than 3 weeks	270 (80.1)	214 (87.3)	0.02
Coughing up blood	272 (80.7)	230 (93.9)	<0.001
Weight loss	201 (59.6)	205 (83.7)	<0.001
Fever or swetting	138 (40.9)	176 (71.8)	<0.001
Chest pain	49 (14.5)	170 (69.4)	<0.001
Shortness of breath	185 (54.9)	225 (91.8)	<0.001
Weakness or loss of apatitt	107 (31.8)	197 (80.4)	<0.001
Rash	46 (13.6)	61 (24.9)	0.07
Do not Know	17 (5.0)	6 (2.4)	0.39
Do you Know how a person could get TB			
Yes	293 (87.2)	242 (98.0)	<0.001
No	43 (12.8)	5 (2.0)	0.24
Do you know how TB is transmitted Coughing and Sneezing of a person having TB	149 (44.2)	64 (25.9)	0.01
TB is curable			
Yes	325 (97.0)	247 (100)	<0.001
No	10 (3.0)	0 (0.0)	-
Treatment			
DOTs			
Yes	179 (53.3)	128 (51.8)	0.39
No	157 (46.7)	119 (48.2)	0.39
Specific drugs provided by health facility			
Yes	122 (36.3)	186 (75.3)	<0.001
No	214 (63.7)	61 (24.7)	<0.001
Do not Know	25 (7.4)	1 (0.4)	0.55
Do you know that the drugs are available for free			
Yes	204 (60.4)	241 (97.6)	<0.001
No	134 (39.6)	6 (2.4)	
PTB is preventable			
Yes	332 (95.8)	245 (99.6)	<0.01
No	14 (4.2)	1 (0.4)	
Covering mouth and nose while coughing/sneezing	243 (72.5)	235 (95.1)	<0.001
Overall TB knowledge Assessment			
Low overall TB Knowledge (<50%)	42 (12.4)	2 (0.8)	0.30
High overall TB Knowledge (>50%)	296 (87.6)	245 (99.2)	<0.001
Knowledge about Bacilli as causes of TB	216 (63.9)	200 (81)	<0.001
Knowledge about signs and symptoms			
Low Knowledge (<50%)	138 (40.9)	35 (14.3)	<0.001
High Knowledge (>50%)	199 (59.1)	210 (85.7)	<0.001

Most (87.6% pastoralists vs. 99.2% sedentary group, p<0.001) had high overall TB knowledge. The Pastoralists had a lower overall TB knowledge (COR = 0.057, 95% CI = 0.0.14–0.24) compared to the sedentary group and their overall TB knowledge decreased by 94% among the pastoralists after adjusting for frequency of health care facility visit (AOR = 0.06, 95%CI = 0.12–0.29) and first heard about TB from HP/HEWs (AOR = 0.14, 95% CI = 0.03–0.61) compared to the sedentary group **(Table 4)**.

**Table 4:** Factors associated with high overall TB knowledge among the pastoralists and sedentary communities of Fentale district, Ethiopia.

TB knowledge	Model 1		Model 2		Model 3		Model 4		Final model	
Co variates	COR(95% CI)	P-value	AOR(95%CI)	p-value	AOR(95%CI)	p-value	AOR(95%CI)	p-value	AOR(95%CI)	p-value
Sedentary	Reference		Reference							
Pastoralists	0.057(0.01–0.02)	<0.01	0.07(0.02–0.29)	<0.01	0.06(0.12–0.29)	<0.01	0.14(0.03–0.61)	0.01	0.18(0.34–0.98	0.048
Duration of stay			1.06(1.03–1.09)	<0.01					1.06(1.03–1.10)	<0.01
Frequency of HCF visit										
Twice a year					Reference					
Once a year					0.36(0.12–1.1)	0.06			0.27(0.07.1.04)	0.06
At least twice in the last 2 years					1.33(0.16–10.96)	0.79			0.87(0.16–7.8)	0.91
Once in the past 5 years					0.09(0.02–0.32)	<0.01)			0.95(0.22–0.42)	<0.01
Never					0.15(0.07–0.34)	<0.01			0.11(0.5–0.3)	<0.01
First heard about TB FROM hcp/HEW’s										
No							Reference			
Yes							4.85(1.9–12.3)	<0.01	3.4(1.3–9.5)	0.02

### Attitude towards TB

Most respondents (95.3% pastoralists vs. 91.1% sedentary group, p = 0.02) mentioned TB as a very serious disease **(Table 5)**. Also (45.3% pastoralists vs. 39.7% sedentary group, p = 0.06) mentioned that they would experience fear if they find out they had TB and (58.3% pastoralist vs. 77.3% sedentary group, p<0.001) mentioned that they would discuss their illness with their spouse if they had TB. Few pastoralists compared to the secondary group mentioned that the community would support a person who has TB (46.7% pastoralist vs. 74.1% sedentary group, p<0.01) **(Table 5)**.

**Table 5:** Attitude of the pastoralists and sedentary communities towards TB in Fentale district, Ethiopia.

Question on their attitude		Pastoralists	Settlers	P-value
	Responses	Frequency (%)	Frequency (%)	
In your opinion, how serious a disease is TB?	Very serious	322 (95.3)	225 (91.1)	0.02
How serious a problem do you think TB is in your community?	Very serious	297 (87.9)	214 (86.6)	0.33
What would be your reaction if you find out you have had TB?				
	Feared	153 (45.3)	98 (39.7)	0.21
	Surprised	8 (2.4)	66 (26.7)	0.06
	Ashamed	11 (3.3)	4 (1.6)	0.43
	Embarrassed	102 (30.2)	49 (19.8)	0.09
	Sadness and hopelessness	64 (18.9)	30 (12.1)	0.20
Who would you talk to about your illness if you had TB?				
	Spouse	197 (58.3)	191 (77.3)	<0.001
	parent	112 (33.1)	36 (14.6)	0.02
	Child/children	0 (0.0)	60 (24.3)	-
	Family member	48 (14.2)	11 (4.5)	0.12
	Close friends	32 (9.5)	5 (2.0)	0.28
	None	5 (1.5)	22 (8.9)	
Do you know people who have/had TB?	Yes	274 (81.1)	241 (97.6)	<0.001
In your community, how is a person with TB usually regarded/treated?				
	Most people reject him/her	135 (39.9)	22 (8.9)	<0.001
	Most people are friendly but try to avoid him/her	38 (11.2)	38 (15.4)	0.29
	The community mostly support him/her	158 (46.7)	183 (74.1)	<0.001
	Others	7 (2.1)	4 (1.6)	0.47
Do you think that HIV + people should be concerned about TB?	Yes	199 (58.9)	215 (87.0)	<0.001
Person with HIV is more likely to develop TB	Yes	180 (90.9)	201 (93.1)	0.11
Person with HIV is not more likely than without HIV to develop TB	Yes	14 (10.1)	11 (34.4)	0.07

### Perceived stigma towards TB

Most respondents mentioned that TB carries less stigma than AIDS (42.9% pastoralists vs. 68.05% sedentary group, p<0.001). The Pastoralists had a higher overall perceived stigma towards TB (61.8%pastoralists vs. 20.2% sedentary group, p<0.001). The Perceived stigma towards TB decreased among the pastoralists by 47% compared to the sedentary group after adjusting for sex (AOR = 0.53, 95% CI = 0.35–0.82), while the perceived stigma score was 11 times higher among the pastoralists after adjusting for sex and educational status of the respondents (AOR = 11, 95%CI = 6.4–18.45) compared to the sedentary communities **(Table 6 and Table 7)**.

**Table 6:** Perceived Stigma towards TB among the pastoralists and sedentary communities in Fentale district, Ethiopia.

Perceived stigma towards TB	responses	Pastoralists	Settlers	P-Value
		Frequency %	Frequency %	
**In your opinion, does TB carry the same stigma as AIDS or less or more**				
	Less	145 (42.9)	168 (68.0)	<0.01
	More	53 (15.7)	51 (20.6)	0.26
	The same	93 (27.5)	28 (11.3)	0.04
	I don’t know	40 (11.8)	0 (0.0)	-
**Overall Perceived stigma (max-11 score)**				
Low perceived stigma (<50%)		128 (38.2)	197 (79.8)	<0.01
High perceived stigma (>50%)		209 (61.8)	50 (20.2)	<0.01
Stigma questiones				
If you had TB, others think less of you		234 (69.2%)	61 (24.7%)	<0.001
If you had TB, you would be ashamed/embarrased		230 (68.0)	90 (36.4%)	<0.001
If you had TB, you would think less of you		203 (60.1%)	35 (14.2%)	<0.001
If you had TB, others would avoid you		245 (72.5%)	66 (26.7%)	<0.01
If you had TB, it would be a problem to find a partner for marriage		150 (44.4%)	51 (20.6%)	<0.001
If you had TB, your partner would refuse to have sex with you		170 (50.3%)	92 (37.2%)	0.02
If you had TB, you would be asked to stay away from social groups		161 (47.6%)	91 (36.85)	0.048
If you had TB, do you think you would disclose your status		218 (64.5%)	212 (85.8%)	<0.001
If you had TB, you would feel like affected by serious disease		41 (12.1%)	14 (5.7%)	0.25
If you had TB, others would think less of your family		166 (49.1%)	49 (19.8%)	<0.01
If you had TB, it would be a problem for your children		275 (81.4%)	178 (72.1%)	0.01

**Table 7:** Factors associated with high perceived stigma towards TB among the pastoralists and sedentary communities in Fentale district, Ethiopia.

Perceived Stigma	Model 1	Model 2	Model 3	Final Model
Co variates	Crude OR, (95% CI)	Adjusted OR (95% CI)	Adjusted OR (95% CI)	
Settlers	Reference			
Pastoralists	9.8 (6.36–14.93)	8.7 (5.64–13.40)	9.0 (5.85–13.85)	8.2 (5.3–12.7)
Sex				
Female		Reference		
Male		1.87 (1.2–2.9)		1.76 (1.1–2.7)
TB is transmitted by droplets				
No			Reference	
Yes			2.1 (1.42–3.09)	

### Health care seeking behavior of respondents

When asked how frequently they seek health care from health facilities, 55.1% pastoralists vs. 50.6% sedentary group, p<0.21 answered twice or more per year and (12.4% pastoralists vs. 26.7% sedentary group) mentioned that they seek health care from the clinic or hospital once a year. More than half (56.2% pastoralists vs. 17.8% sedentary group, p<0.001) mentioned that they sought help from THs twice a year or more **(Table 8).**

**Table 8:** Health care seeking behavior of the pastoralists and sedentary communities in Fentale district, Ethiopia.

Health care seeking behavior	Pastoralists (n = 338)	Settlers (n = 247 =	P value
	Frequency %	Frequency %	
**Where do you usually go to seek help for your illness**			
Private clinic	151 (44.7)	141 (57.1)	0.02
Governmental HC/hospital	267 (79.0)	206 (83.4)	0.11
Clinic run by non-governmental Organization/church	2 (0.6)	51 (20.6)	0.24
Traditional healers	48 (14.2)	32 (13.0)	0.41
Religious place	1 (0.3)	19 (7.7)	-
**How often do you generally seek health care at a clinic or hospital**			
Twice a year or more	171 (50.6)	136 (55.1)	0.21
Once a year	42 (12.4)	66 (26.7)	0.04
Less than once a year but at least twice in the past two years	25 (7.4)	21 (8.5)	0.45
Once in the past five years	11 (3.3)	23 (9.3)	0.26
Never	89 (26.3)	1 (0.4)	0.28
**How often do you general seek help from TH’s for your illness**			
Twice a year or more	190 (56.2)	44 (17.81)	<0.001
Once a year	50 (14.79)	62 (25.10)	0.18
Less than once a year but at least twice in the past two years	16 (4.73)	13 (5.26)	0.77
Once in the past five years	7 (2.07)	3 (1.21)	0.74
Never	68 (20.12)	19 (7.69)	<0.001
others	5 (1.48)	24 (9.71)	<0.001

### Focus group discussion

A total of 19 individuals (10 men and 9 women), age range 20–55 years (mean age 35.6 years) participated in the FGD conducted in the Pastoralist community from the *Fentale* districts. Seven discussants (3 male and 4 females), age range 28–55 years (mean age 38.2 years) participated in the FGD held in the sedentary community of *Fentale* districts. All the 19 participants from the pastoralist community mentioned that pastoralism was their main occupation. Among these, 18 were illiterate and 1 has attended primary school. All of them were Muslims and belonged to the Oromo ethnic group. All the 7 discussants from the sedentary group were farmers. Among them, 6 had primary education and 1 had no formal education. All of them were Christians, and 1 belonged to Amhara and Oromo ethnic group, respectively.

According to the discussants from the pastoralist community, TB was mentioned as the second most important public health problem next to malaria, while the sedentary group mentioned TB as the most important public health problem followed by malaria. The pastoralist discussants mentioned cold air, bacteria, dust, environmental and personal hygiene as causes of TB. However, the discussants from the sedentary group mentioned bacteria as the main cause of TB.

All participants from both groups mentioned a persistent cough, cough with sputum, night sweating, weight loss and shortness of breath as the main symptoms of TB. Drugs provided by the health facilities were mentioned as an effective treatment. Almost all the discussants from both groups believe that TB is transmissible and that coughing/sneezing of TB patient and sharing utensils were potential ways of TB transmission.

On the other hand, a 45 years old female discussant from the pastoralist group said “TB is very contagious and it is transmitted from sweat from infected person to uninfected one when they sleep together”.

Covering the mouth and nose during coughing and sneezing were mentioned as ways of preventing TB transmission from both groups. Overcrowding and living with TB patients were mentioned by both groups as a major risk factor for exposure to the disease. All discussants said children and the old ones are the most TB affected members of the community and that is because they cannot keep their own personal hygiene. Most discussants from the pastoralist community and a few from the sedentary group said they would feel ashamed if they found out they have TB.

A 35 years old female pastoralist discussant said “Yes, it is shameful to have TB in our community and we do isolate the patients until they get free from the disease. The only solution for those with TB is to seek treatment from MHFs”.

Almost all participants from both groups said marrying a TB patient is not a problem except one participant from pastoralists who said: *“I will ban the marriage because I don’t want me or my family to suffer from TB”*. All discussants from the pastoralist group mentioned that they would stay away from a person if they know he/she has TB, while discussants from the sedentary group said that they would advise and support him/her if they know he/she has TB.

A 45 years old female discussant among the pastoralists said: “I will not walk or sit near TB patients because the wind could bring the bacteria to me, so I will stay away from the TB patient”.

## Discussion

The results of the present study showed that there is a significant knowledge gap about the causes, signs and symptoms, mode of transmission, prevention, and treatment of TB among the pastoralists compared to their neighboring sedentary population. The proportion of the pastoralists who have indicted bacilli as the cause of TB is lower compared to the sedentary group. It is higher than the studies conducted in Afar and Somali pastoralists and non-pastoral community in Ethiopia [4, 10–12, 15] and elsewhere [17], it is difficult to give a sound reason for the difference but it could be related to the geographical location and sources of information about TB [12, 18]. Rather, most pastoralists in this study have a misconception about the causes of TB, for instance, witchcraft, hard work, and sexual overindulgence were mentioned as the causes of TB. Moreover, misconception about the causes of TB and factors for exposure to the disease, like sharing utensils and unventilated house were reported among most pastoralists as causes of TB compared with sedentary group. These results are in agreement with other studies conducted in Afar and Somali pastoralist [7, 10, 12] and in the non-pastoral community in Ethiopia [16]. For example, studies in Afar and Shinille area pastoralists shows that 38% and 10% respondents mentioned that starvation and poverty as the causes of TB, respectively.

Similarly, fewer pastoralists than the sedentary group mentioned modern drugs as the treatment of TB. This indicates the low TB information coverage about the cause and treatment of TB in the pastoralists community. This could be due to the migratory lifestyle and landscape that makes it difficult to reach them in the routine health education program [7]. Lack of awareness about the causative agent and treatment of TB and traditional beliefs about the causes of TB may have a negative impact on patient attitude towards health care seeking behavior and preventive measures as most people with such belief might not visit health care facilities or may consider traditional methods. This in turn, could result in prolonged diagnostic and treatment delay and contribute to the spread of disease in the pastoralist community [7, 8, 11, 12, 16].

Likewise, few pastoralists heard about TB mainly from the health professionals and/ or health extension workers compared to the sedentary group. The difference in knowledge might be related to the frequency of contact with HCP/HEW’s and ineffective communication strategy of the national TB control program which doesn’t consider the pastoralists way of life. This is in accordance with other studies in the pastoralists community in Ethiopia [4, 12] and in Pakistan [18]. For instance, the study in the Shinille area in the Somali Region of Ethiopia has shown that only 33% of respondents mentioned health service providers as TB information source. This indicates that the link between the HCP/HEW’s and the pastoralists is weaker than the sedentary community. The Multivariable logistic regression analysis showed that being pastoralists were less likely to be predictive of high overall TB knowledge compared to being sedentary. This is in line with the studies done in the other pastoralist area in Ethiopia [12, 19]. For example, in a study done among pastoralists and agro-pastoralists of the middle and lower rift Valley of Afar region, agro-pastoralism as an occupation was a predictive of high biomedical overall knowledge of PTB than nomadic pastoralism [10]. Moreover, the length of stay in the study area, the frequency of health facility visits and sources of information were predictors of overall TB knowledge.

The Pastoralists considered TB as a very serious disease in general and a very serious problem in that specific community compared to the sedentary group, in addition, the majority of the pastoralists in this study expressed that they would feel fear and embarrassed if they found out they have TB and the community mostly rejects TB patients compared to the sedentary group. Similar feelings have been reported in previous studies in the pastoralist communities [12] and elsewhere [11, 18, 20]. Personal stigma because of the attitude towards TB may result from a fear of transmitting infection, perceived causes of TB or avoiding potential discrimination in the community, while community stigma may result from the perceived risk of infection and link between TB and the perceived causes of TB [18]. For example, witchcraft was mentioned by most respondents in the pastoralists group as the causes of TB in this study. It might also be because of its mortality in the community, the chronic signs, and symptoms of the disease like a persistent cough with blood and the long-term treatment needed to cure TB[11]. Lack of basic awareness about TB is an important factor in attitude towards TB, which might lead to negative attitudes towards TB status exposure, health seeking behavior and adherence to TB treatment.

The majority of the pastoralists in this study had a high overall perceived stigma towards TB and few pastoralists also mentioned that they would disclose their status if they had TB compared to the sedentary group. TB stigma was expressed like, they would think less of themselves, they would be ashamed/ embarrassed and also mentioned the perceived stigma of TB on marital status, sexual and social relations and the problem of their children and this was supported by the results of FGD. Most pastoralist discussants mentioned that they would sit at a distance from a TB patient to avoid any chance of infection. A report from the study done in the Gilgel Gibe area of Southwest Ethiopia also supported this finding [16]. We also identified that being a pastoralist, being male and knowing that TB is transmitted by droplets from coughing or sneezing of active TB patient were predictors of high overall perceived stigma towards TB. The Multivariable logistic regression analysis showed that the overall perceived stigma is nine times higher among the pastoralists compared to the sedentary group after controlling the droplets from coughing and sneezing of active TB patient as a route of TB transmission, which could be related to the perception about the contagiousness of TB. Such perceptions might have a serious impact on the emotional, psychological and social wellbeing of the TB patients and their family. This indicates that the stigma towards TB patients in the pastoralist community could lead people to the sign and symptoms of having negative attitude towards healthcare seeking practice and hiding their signs and symptoms to avoid the discrimination which could lead to the spread of the disease and the delayed diagnosis and treatment [12, 16, 18].

The Lack of awareness about the cause and treatment of TB has been long associated with the poor health care seeking behavior and delayed diagnosis and treatment because of the first consultation of traditional healers or non-health professionals [4, 9, 17, 21]. The majority of pastoralists in this study mentioned that they sought help for their illness from traditional healers twice or more per year. This might be because of the poor health care access in the pastoralist community [4]. The low access to health care can be explained as no access to health facility due to long distance and/or poor access and costs of transportation to health facility which is a serious problem in the pastoralist community [19] and rural areas as the whole of Ethiopia [20]. For instance, a study conducted in the Somali region, the pastoralists reported that TB patients preferred traditional healers because of easy access and the long distance to heath care facility [7]. Also, traditional beliefs about the causes of TB, like witchcraft, might lead the community to consider traditional healing and avoid health care facility visit or default from treatment considering the modern treatment ineffective for such cause[22].

### Limitations

The Knowledge, attitude and perceived stigma of the pastoralist community were assessed in comparison to their neighbor the sedentary community and so far this is the first time to conduct a comparative study between the pastoralists and sedentary communities in Ethiopia. This could help to develop TB control strategy pertinent to the pastoralist way of life. However, the study has limitations because the report does not include the status of TB infection and disease in the communities studied. Also, the stigma questionnaire was not validated.

## Conclusions and recommendations

We found that TB is familiar to both the pastoralist and sedentary communities in this study area. However, the pastoralists had a higher knowledge gap about TB, such as the cause of TB, a negative attitude such as feeling fear or embarrassment if had TB, high overall perceived stigma and a more frequent visit to TH’s for their illness compared to the sedentary community. The existing TB control strategy is not working the same way for the pastoralist compared to the sedentary community in the raising awareness about TB. To overcome this challenge, new TB control strategy involving traditional healers and volunteer CHW’s should be adapted for the pastoralist community which could be pertinent to their lifestyle. Health education interventions and social mobilization by integrating and training traditional healers/CHW’s should be considered to address the traditional beliefs about the causes of TB, to avoid unfavorable attitude towards TB, to avoid causes of stigma and increase health care seeking behavior in the pastoralist community.

## Supporting information

S1 File Questionnaire. (DOCX)
