# Supplementary material for: Knowledge, attitude and perceived stigma towards tuberculosis among pastoralists; Do they differ from sedentary communities? A comparative cross-sectional study
Source: PLoS One. 2017 Jul 17;12(7):e0181032. doi: 10.1371/journal.pone.0181032 (PMC5513437; doi:10.1371/journal.pone.0181032)
Supplement: S1 File — (DOCX) [file pone.0181032.s001.docx]

**Questionnaire for community based cross sectional study on Knowledge, attitude and perceived stigma towards TB and health seeking behavior among Pastoralist and Sedentary communities of *Fentale* District.**

**Part I. Socio- demographic characteristics of the respondents**

1. ID number of respondent_________________________________
2. District ____________________________Kebele__________________
3. Village________________________________- House No._____________
4. Sex : 1= Male 2= Female
5. Age (Year): ____________
6. Ethnicity: 1= Oromo 2= Amhara 3=other (specify) ________________________________
7. Religion 1= Muslim 2= Christianity 3= other specify _________________________________
8. Marital status 1= Married 2= single 3 = divorced 4= widowed
9. Educational status: 1= can’t read & write 2= read & write 3= primary (1-8) 4= secondary (9-12) 6= other (specify)_______________________-
10. Occupation: 1= Nomadic pastoralist 2= Agro-pastoralist 3= farmer 4= Business 5= Civil servant 6= student 7= other (specify) ____________________________
11. Duration of residence in the area/village _______________ (years)
12. Do you migrate in dry Seasons?

1= Yes 2= No

1. If yes, where do you migrate most of the time?

1= within the district 2= outside the district

1. Frequency of migration: per year: __________
2. Family members who migrates mostly

1= head of the household

2= children

3= all family members

4= other (specify)_________________

**Part II. Assessments of knowledge about PTB**

1. Have you ever heard about the disease called PTB?

1= Yes 2= No

1. If yes, from whom?

1= From friend (s) 2= From health workers /Health extension workers

3= From media (radio, TV poster etc) 4= from patient 5= family member/friends

6= Other (specify): __________________________

1. What do you think is the cause of PTB? **(Check all that are mentioned)**

1= Bacteria/germ 2= witchcraft 3= shortage of food 4= unventilated house 5= malnutrition 6= sexual overindulgence 7= hard work 8= poverty 9= living together with untreated TB patient others (specify):_____________________

1. What are some of the common symptoms of TB? **(Check all that are mentioned)**

1= cough 2= Cough for 3 or more weeks 3= cough up blood 4= weight loss

5= shortness of breath 6= Fever/sweat at night 6= chest pain 7= weakness or loss of appetite 8= rash 9= other (Specify)_____________ 10= do not know

1. Do you know how a person could get TB?

1= Yes 2= No

1. If yes to Q7, How can a person get TB? **(Check all that are mentioned)**

1= through droplets from coughing or sneezing of a person having TB

2= hand shaking of a person having TB 3= kissing TB infected person

4= drinking raw milk 5= eating TB infected meat

6= exposure to cold air 7= sharing cup with a person having TB

8= sharing utensils 9= chewing chat together

10= Others (specify) :______________

1. Who do you think can transmit TB? **(Check all that are mentioned)**

1= a person who have active TB and not taking TB drugs

2= Anyone who has cough

3= a person who is diagnosed having TB and finished treatment

4= others specify

1. Do you think that the transmission of PTB can be prevented?

1= Yes 2= No

1. If Yes to Q12, How can TB be prevented **(Check all that are mentioned)**

1= using separate room 2= covering mouth and nose while coughing or sneezing

3= avoiding sharing cups 4= avoiding eating raw meat

5= avoiding drinking raw milk 6= through good nutrition

7= by prayer 8= others (specify):__________________

1. In your opinion, who can be infected with TB? **(Check all that are mentioned)**

1= anybody 2= Poor people 3= homeless people 4= only alcoholics

5= only people living with HIV 6= Others (specify): __________

1. Can TB be cured?

1= Yes 2= No

1. If Yes to Q15, How can someone with TB be cured? **(Check all that are mentioned)**

1= traditional Medicine 2= home rest without medicine 3=praying

4= specific drugs given by health facility 5= DOTS

6= do not Know 7= others (specify): _____________

1. Where can TB be cured? **(Check all that are mentioned)**

1= public health facility 2= by visiting traditional healers

3= in Religious places others (specify):______________

1. Do you know that the drugs are available for free?

1= Yes 2= No 3= I don't know

**Part III. Assessments of Sources of TB information**

1. Where did you first heard about PTB?

1= from friend (s) 2= from health workers /Health extension workers 3= From media (radio, TV poster etc) 4= from patient 5= family member/friends

6= other (specify): ___________________________

1. Is information on TB available to you?

1= Yes 2= No 3= I don’t know

1. What is the most trusted sources of health information for you? **(Check all that are mentioned)**

1= health professionals/Health extension workers

2= family/Friends

3= mass media (TV, radio)

4= religious leaders

5= others (specify):__________

### Part III: Assessment of respondents attitude towards Tuberculosis

1. In your opinion, how serious a disease is TB?

1= Very serious

2= somewhat serious

3: Not very serious

1. How serious problem do you think TB is in your community?

1= Very serious

2= somewhat serious

3= Not very serious

1. What will be your reaction if you found out that you have TB? **(Check all that are mentioned)**

1= Feared 2= Surprised 3= Ashamed 4=Embarrassed

5= Sadness and Hopeless

1. If you found out you have TB, Whom would you talk to about your illness? **(Check all that are mentioned)**

1= Spouse 2= Parent 3= Child/Children 4= other family member

5= Close friends 6= No one Others (specify=: __________________

1. In your community, how is a person with TB usually regarded/treated?

1= most people reject him or her

2= most people are friendly, but they generally try to avoid him/her

3= the community mostly supports and help him/her

4= others please specify:

1. Do you think that HIV positive people should be concerned about TB?

1= Yes 2= No

If yes, why?

1= Person with HIV is more likely to develop TB

2= Do not know

3= Others (Specify):

1. In your opinion, does having TB carry the same stigma as AIDS or less/more

1= less 2= more 3= the same

4= others (specify) : _

**Part IV: Assessments of perceived stigma towards TB**

- 1. If you had TB, others will think less of you?

1= Yes 2= No 3= Do not Know

- 1. If you find out you have TB, you would feel ashamed and embarrassed?

1= Yes 2= No 3= Do not know

- 1. If you had TB, you would think les of you?

1= Yes 2= No 3= I don’t know

- 1. If you had TB, others would avoid you?

1= Yes 2= No 3= I don’t know

- 1. If you had TB, you would have a problem of finding a partner for marriage even after the cure?

1= Yes 2= No 3= I don’t know

- 1. If you had TB, your partner would refuse to have sex with you?

1= Yes 2= No 3= I don’t know

- 1. If you had TB, you would be asked to stay away from social groups?

1= Yes 2= No 3= I don’t know

- 1. If you had TB, do you think you would disclose your status to anyone?

1= Yes 2= No 3= I don’t know

- 1. If you had TB, you would feel like you would make others affected by the disease?

1= Yes 2= No 3= I don’t know

- 1. If you had TB, you would feel like others would think les of your family?

1= Yes 2= No 3= I don’t know

- 1. If you had TB, do you think it would be a problem to your children?

1= Yes 2= No 3= I don’t know

**Part VI: Assessment of health seeking behavior of respondents**

1. Where do you usually go if you are sick or to treat a general health problem? (Check all that are mentioned)

1= Private clinic

2= Government clinic or Hospital

3= Traditional healer

4= Clinic run by nongovernmental organization or church

5= Religious places

6= others (specify):

1. How often do you generally seek help for your illness from health facility? (check one)

1= twice a year or more

2= once a year

3= less than once a year but at least twice in the past two years

4= once in the past two years

5= never in the past five years

6= others please specify: ____________

1. How often do you generally seek help for your illness from traditional healers? (check one)

1= twice a year or more

2= once a year

3= less than once a year but at least twice in the past two years

4= once in the past two years

5= never in the past five years

6= others please specify:

1. If you would not go to the health facility, what is the reason? **(Check all that are mentioned)**

1= Not sure where to go

2= Cost

3= Do not trust health workers

4= Do not like attitude of health workers

5=cannot leave work

6=Others (specify): ________________
